# The positive relationship between the Alternative Healthy Diet Index and serum klotho levels: A cross-sectional analysis in middle-to-older Americans

**DOI:** 10.1371/journal.pone.0323228

**Published:** 2025-05-07

**Authors:** Chaoshun Zheng, Longsheng Zhang, Yueyue Guo, Chuchun Lin, Xuhui He

**Affiliations:** 1 Department of Orthopedics II, Jieyang People’s Hospital, Jieyang, China; 2 Department of anesthesiology, Jieyang People’s Hospital, Jieyang, China; Universitas Airlangga Fakultas Kedokteran, INDONESIA

## Abstract

**Background and aim:**

The Alternative Healthy Eating Index (AHEI-2010) is a dietary index associated with chronic diseases and serves as an important metric for assessing the healthiness of one’s diet. Serum Soluble Klotho (S-Klotho) is a protein related to anti-aging. There is currently a lack of research on the relationship between AHEI and S-Klotho. This study evaluated the relationship between the AHEI index and S-Klotho in the U.S. middle-aged and elderly population from the National Health and Nutrition Examination Survey (NHANES) 2011–2016.

**Methodology:**

This study includes 6,305 middle-aged and elderly participants (aged 40–79 years old). The interrelationship between the AHEI-2010 and S-klotho concentration was explored using multivariate regression models, and the nonlinear relationship between the two was investigated through curve fitting. The stability of this relationship in different populations was explored through subgroup analysis.

**Results:**

There was a positive correlation between AHEI and S-klotho (β=1.1, 95% CI: 0.1, 2.1, P = 0.035). The multivariate-adjusted β and 95% confidence intervals (CIs) from the lowest to the highest AHEI-2010 categories (＜31.9, 31.9–39.3, 39.3–47.9, and > 47.9) were 0.0 (reference), 15.7 (-13.5, 44.9), 12.5 (-16.5, 41.6), and 31.9 (2.9, 60.9), respectively. The curve fitting found that the relationship between AHEI-2010 and S-Klotho is essentially linear, with S-Klotho levels increasing linearly as AHEI improves.

**Conclusion:**

AHEI is positively associated with S-Klotho levels in American middle-to-older adults. Further research is needed to elucidate the causal relationship and specific mechanisms.

## 1. Introduction

Aging is an inevitable, complex, and multifaceted process of life, essentially characterized by systemic deterioration at the organ, tissue, and cellular levels, leading to serious consequences such as chronic diseases and disability [1]. The accelerated aging of the population and the associated social burden have placed aging at the center of scientific research, and the fight against aging has always been an important topic in the scientific field. Strategies against aging are expected to delay, stop, and even reverse the aging process, extending the length of a healthy life. Currently, the main strategies confirmed effective in experimental models to combat aging include exercise, nutrition, genetic and pharmacological interventions, suggesting that they may delay or prevent aging in humans [2]. Diet, as an important aspect of anti-aging, has been proven to reduce age-related diseases such as sarcopenia, cognitive decline, osteoporosis, hearing loss, and urinary incontinence [3].

Klotho, as one of the biomarkers related to aging, has been proven to have a decreased level associated with a shorter lifespan [3]. The name Klotho is derived from the Greek goddess “Clotho” symbolizing its ability to regulate metabolism and extend the length of life. Klotho proteins primarily exist in two forms within the body: membrane Klotho and soluble Klotho, which have different functions [4]. Membrane Klotho forms a complex with fibroblast growth factor (FGF) receptors, promoting phosphorus metabolism into urine[4]. S-Klotho in the plasma exerts systemic effects on multiple organs. As age increases, S-Klotho levels decrease [5]. High levels of S-Klotho have been proven to protect vital functions such as the heart, kidneys, bones, and cognition [6–9], and it has now become an important target in the fight against aging.

The AHEI is an important dietary index designed to incorporate a variety of factors that may lead to chronic diseases. It has been proved to be correlated with the incidence of various chronic diseases such as coronary heart disease, diabetes, stroke, and hypertension [10], meaning that a higher AHEI score is associated with a lower incidence of these diseases. However, there is currently a lack of research on the relationship between this index and S-Klotho. This study aims to explore the impact of AHEI on S-Klotho levels in the American population through NHANES, providing a theoretical basis for the diagnosis and treatment strategies of aging.

## 2. Materials and methods

### Study population

This study utilizes publicly available data from the National Health and Nutrition Examination Survey (NHANES) (https://wwwn.cdc.gov/nchs/nhanes/). The NHANES database is conducted by the National Center for Health Statistics (NCHS) of the Centers for Disease Control and Prevention (CDC). It is an important health and nutritional status survey program that began in the last century. Since 1999, NHANES has become a continuous project, collecting data in two-year cycles and obtaining data representative of the United States through random sampling methods. The statistics include demographic data, dietary data, physical examination data, laboratory data, and questionnaire data, providing the foundation for the formulation of public health decisions.

**Fig 1:**
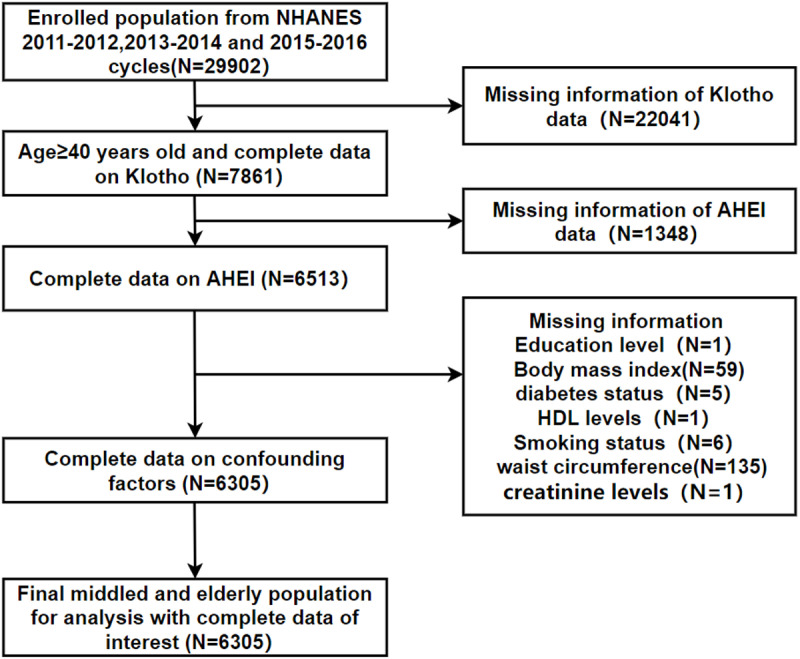
Flow diagram of inclusion criteria and exclusion criteria.

In this study, we selected data from three cycles between 2011 and 2016 for research. The survey subjects were middle-aged and elderly individuals over the age of 40, with complete S-Klotho data and dietary survey data (capable of calculating the AHEI index). Individuals with missing confounding factors were excluded, such as those lacking in education level, Body mass index (BMI), diabetes status, High-Density Lipoprotein (HDL) levels, smoking status, waist circumference, and creatinine. The details of inclusion and exclusion is shown in Fig 1. The studies involving human participants were reviewed and approved by the National Center for Health Statistics, Research Ethics Review Board (ERB). The patients/participants provided their written informed consent.

### S-Klotho and AHEI

S-Klotho analyses were performed with a commercially available ELISA kit (IBL International, Japan). Available pristine serum samples were taken from 40–79 years old participants and analyzed with the IBL ELISA method [7,11]. All sample analyses were performed twice to ensure the accuracy of the test. Those with duplicate results exceeding 10% were flagged for analyses to be repeated.

The dietary quality in this study was assessed using the Alternative Healthy Eating Index (AHEI) score, which is based on foods and nutrients that may predict the risk of chronic diseases [10]. It includes a total of 11 components, such as vegetables, fruits, whole grains, nuts and legumes, long-chain omega-3 fatty acids, polyunsaturated fatty acids, sugar-sweetened beverages and fruit juices, red and processed meats, trans fatty acids, sodium, and alcohol. The total score is 110 points, with a higher score indicating a healthier diet, which is more effective in assessing the risk of chronic diseases [3], and is closely related to all-cause mortality, cardiovascular disease mortality, and cancer mortality[12]. Given that the components of the AHEI are derived from common dietary components of populations with diverse ethnicities, lifestyles, and living environments, we believe that it is feasible and universally applicable to adjust dietary structures based on AHEI scores. Moreover, the multi-ethnic composition of our study population effectively demonstrates the feasibility of promoting the AHEI across different demographic groups.

In this article, the raw data is derived from two 24-hour dietary recalls. The AHEI score was calculated using statistical packages R (The R Foundation; version 4.4.1) and the Dietaryindex package based on the raw data [13] The Dietaryindex is an R package offering streamlined, user-friendly approaches for the standardization of compiling dietary intake information into index-based dietary patterns, facilitating the evaluation of adherence to these patterns in both epidemiological and clinical research studies [13]. The Dietaryindex package was also used in research related to dietary indices [14].

### Covariates

Possible confounding factors include the following:

Demographic characteristics such as age, gender (male, female), race/ethnicity (Mexican Americans, other Hispanic people, non-Hispanic White people, non-Hispanic Black people, other race), educational level (less than high school, high school or equivalent, College or above), and Family poverty income ratio (<1.3, 1.3–3.5, > 3.5, not recorded) were considered confounding factors in this study and were used for adjustment in statistical models.

Based on relevant research and clinical experience, other related factors were also used in this study. These include smoking status (≥100 in life, < 100 in life), drinking status (≥12 per year, < 12 per year, not recorded), hypertension status (yes, no, not recorded), diabetes status (yes, no, borderline), BMI, waist circumference (cm), physical activity (≥600 METs(Metabolic Equivalent) per week, < 600 METs per week, not recorded), energy intake level (Kcal/day), HDL level (mmol/L), and creatinine level (mg/dL), all of which are incorporated into the relevant models. In terms of energy intake, we used the average data from the two-day retrospective analysis. If there is only one day’s data available, that data is used as the statistical data.

### Statistical analysis

All statistics in this study were conducted using a weighted method to ensure that the results are representative of the whole United States, following the CDC guidelines [15]. The AHEI index was divided into four groups based on quartile points, and the statistical characteristics of each group were described separately, with the lowest quartile group serving as the control group. Categorical variables were represented by frequency (percentage) and compared between groups using the chi-square test. Continuous variables were expressed as the mean ± standard deviation and compared between groups using the independent samples t-test.

To study the linear relationship between the exposure factor (AHEI score) and the outcome factor (S-Klotho level), we used a single-factor linear regression equation and constructed three models. Model one was the unadjusted model, model two adjusted for age, sex, and race/ethnicity, and model three further adjusted for education level, Poverty Impact Ratio (PIR), drinking status, smoking status, BMI, diabetes, hypertension, HDL, physical activity, waist circumference, energy intake level, and creatinine level.

**Fig 2:**
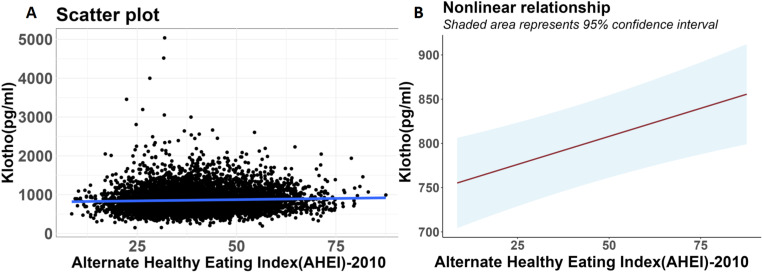
Relationship between AHEI-2010 and S-Klotho. **(A)** Each black dot represents a sample. The blue solid line indicates the linear fitting result between the two. **(B)** The red solid line represents the smooth curve fitting between the two variables. The blue shaded area represents the fit’s 95% confidence interval. Adjusted for age, sex, race/ethnicity, education level, PIR, drinking status, smoking status, BMI, diabetes, hypertension, HDL, physical activity, waist circumference, energy intake level, and creatinine level.

To explore the nonlinear relationship between the exposure and outcome factor, we used smooth curve fitting for analysis (adjusted for age, sex, and race/ethnicity, and model three further adjusted for education level, Poverty Impact Ratio (PIR), drinking status, smoking status, BMI, diabetes, hypertension, HDL, physical activity, waist circumference, energy intake level, and creatinine level) and plotted Fig 2. Finally, stratified analyses were conducted by gender, age, and race/ ethnicity. The P for interaction was calculated to determine whether there are significant differences in this relationship across different subgroups. The relevant statistics were performed using the Log likelihood ratio test method, which assesses the significance of the interaction by comparing two regression models—one with and one without the interaction term.

All analyses were performed using statistical packages R (The R Foundation; version 4.4.1) and EmpowerStats (X&Y Solutions Inc.), with a p-value of less than 0.05 considered to indicate statistical significance.

## 3. Results

### Characteristics of participants

We obtained 29,902 participants from three cycles (NHANES 2011–2012, 2013–2014, 2015–2016). By setting inclusion and exclusion criteria, we ultimately recruited 6,305 eligible participants, with the specific screening process shown in Fig 1. The data were divided into four groups based on the quartiles of AHEI levels and their basic characteristics were described and compared between groups, as seen in Table 1. Compared to the lowest quartile group, the highest quartile group was older, had a higher proportion of females, more Non-Hispanic white people, higher education levels and PIR levels, higher rates of non-smokers and non-drinkers, were physically active, had lower prevalence of diabetes and hypertension, lower BMI, smaller waist circumference, higher HDL levels, lower creatinine levels and had higher S-Klotho levels, but higher energy intake levels (p < 0.05).

**Table 1:** Characteristics according to AHEI quartile.

	Q1 ＜31.9	Q2 31.9-39.3	Q3 39.3-47.9	Q4 ＞47.9	P value
Age(years)	54.4 ± 9.9	56.9 ± 10.4	57.0 ± 10.6	57.9 ± 9.9	＜0.001
Gender(%)					＜0.001
Male	51.8	47.5	47.8	40.9	
Female	48.2	52.5	52.2	59.1	
Race/ethnicity (%)					＜0.001
Mexican American	5.6	7.6	6.9	4.2	
Other Hispanic	6.0	5.1	4.9	3.7	
Non-Hispanic white people	70.2	73.7	75.3	77.9	
Non-Hispanic black people	13.3	8.6	7.5	5.5	
Other	5.0	5.1	5.5	8.7	
Education (%)					＜0.001
Less than high school	5.1	5.9	4.6	2.7	
High school or equivalent	45.0	33.9	26.6	16.0	
College or above	49.9	60.2	68.8	81.4	
Family poverty income ratio (%)					＜0.001
<1.3	24.5	17.6	12.4	8.0	
1.3–3.5	40.2	34.5	29.5	23.3	
>3.5	30.7	42.1	52.4	62.1	
Not recorded	4.6	5.7	5.7	6.6	
Drinking status(%)					0.001
＜12 alcoholic drinks/per year	75.1	74.5	76.5	79.2	
≥ 12 alcoholic drinks/per year	21.4	22.5	20.6	16.7	
Not recorded	3.5	2.9	3.0	4.0	
Smoking status(%)					＜0.001
≥100 cigarettes in life	57.8	48.6	46.0	40.4	
＜100 cigarettes in life	42.2	51.4	54.0	59.6	
PHYSICAL.ACTIVITY((MET-min per week)					＜0.001
＜600	40.4	35.9	41.0	43.7	
≥600	30.3	34.4	35.7	42.1	
Not recorded	29.3	29.7	23.4	14.2	
Diabetes (%)					0.028
Yes	13.9	15.5	14.4	11.5	
No	82.9	82.0	83.1	85.5	
Borderline	3.3	2.5	2.5	3.0	
Hypertension					＜0.001
Yes	46.0	45.7	39.8	36.0	
No	54.0	54.1	60.0	64.0	
No recorded	0.0	0.2	0.1		
Body mass index (kg/m^2^)	30.9 ± 6.9	30.4 ± 6.7	29.9 ± 6.4	27.9 ± 5.9	＜0.001
high density lipoprotein (mmol/L)	1.3 ± 0.4	1.4 ± 0.5	1.4 ± 0.4	1.5 ± 0.5	＜0.001
Waist circumference (cm)	105.4 ± 15.9	104.4 ± 15.7	102.7 ± 15.2	97.7 ± 14.2	＜0.001
Energy intake (kcal/day)	1964.7 ± 759.7	1994.8 ± 760.4	2047.1 ± 738.9	2119.5 ± 691.8	＜0.001
Creatinine(μmol/L)	81.6 ± 31.5	80.3 ± 37.7	79.6 ± 36.6	76.1 ± 22.7	＜0.001
S-Klotho(pg/ml)	831.9 ± 331.3	843.9 ± 290.7	842.3 ± 289.1	865.6 ± 268.0	0.010

#### 3.1. Linear relationships between AHEI-2010 and S-Klotho.

We explored the linear relationship between AHEI-2010 and S-Klotho using multiple linear regression analysis and constructed three models for verification. The specific results are shown in Table 2. The linear regression relationship between AHEI and S-Klotho in the three models is statistically significant (P < 0.05). To further verify the relationship between the two, we categorized AHEI according to the quartile grouping. After adjusting for multiple factors (age, sex, race/ethnicity, education level, PIR, drinking status, smoking status, BMI, diabetes, hypertension, HDL, physical activity, waist circumference, energy intake level, and creatinine level), the multivariate-adjusted β and 95% confidence intervals (CIs) from the lowest to the highest AHEI-2010 categories (＜31.9, 31.9–39.3, 39.3–47.9, and > 47.9) were 0.0 (reference), 15.7 (-13.5, 44.9), 12.5 (-16.5, 41.6), and 31.9 (2.9, 60.9), respectively. The p for trend is less than 0.05, indicating an increasing trend in S-Klotho values with the improvement of AHEI categories.

**Table 2:** Relationship between Alternate Healthy Eating Index (AHEI)-2010 and S-Klotho.

Exposure	Non-adjusted	Adjust I	Adjust II
AHEI	1.3 (0.5, 2.1) 0.001	1.7 (0.9, 2.5) <0.001	1.1 (0.1, 2.1) 0.035
AHEI categories			
＜31.9	Reference	Reference	Reference
31.9–39.3	12.1 (-18.0, 42.2) 0.432	21.6 (-8.4, 51.6) 0.159	15.7 (-13.5, 44.9) 0.293
39.3–47.9	10.4(-18.7,39.5) 0.482	21.5(-7.5, 50.4) 0.146	12.5 (-16.5,41.6) 0.397
>47.9	33.7 (5.7, 61.8) 0.018	47.0 (18.8,75.2) 0.001	31.9(2.9, 60.9) 0.031
P for trend	0.020	0.001	0.044

#### 3.2. Non-linear relationships between AHEI-2010 and S-Klotho.

To explore the nonlinear relationship between AHEI-2010 and S-Klotho, we plotted a scatter plot (Fig 2A). After adequate adjustment (Adjusted for age, sex, race/ethnicity, education level, PIR, drinking status, smoking status, BMI, diabetes, hypertension, HDL, physical activity, waist circumference, energy intake level, and creatinine level

), we used curve fitting to analyze the nonlinear relationship between the two and found that the relationship between AHEI-2010 and S-Klotho is essentially linear, with S-Klotho levels increasing linearly as AHEI improves (Fig 2B).

#### 3.3. Stratified analyses.

**Fig 3:**
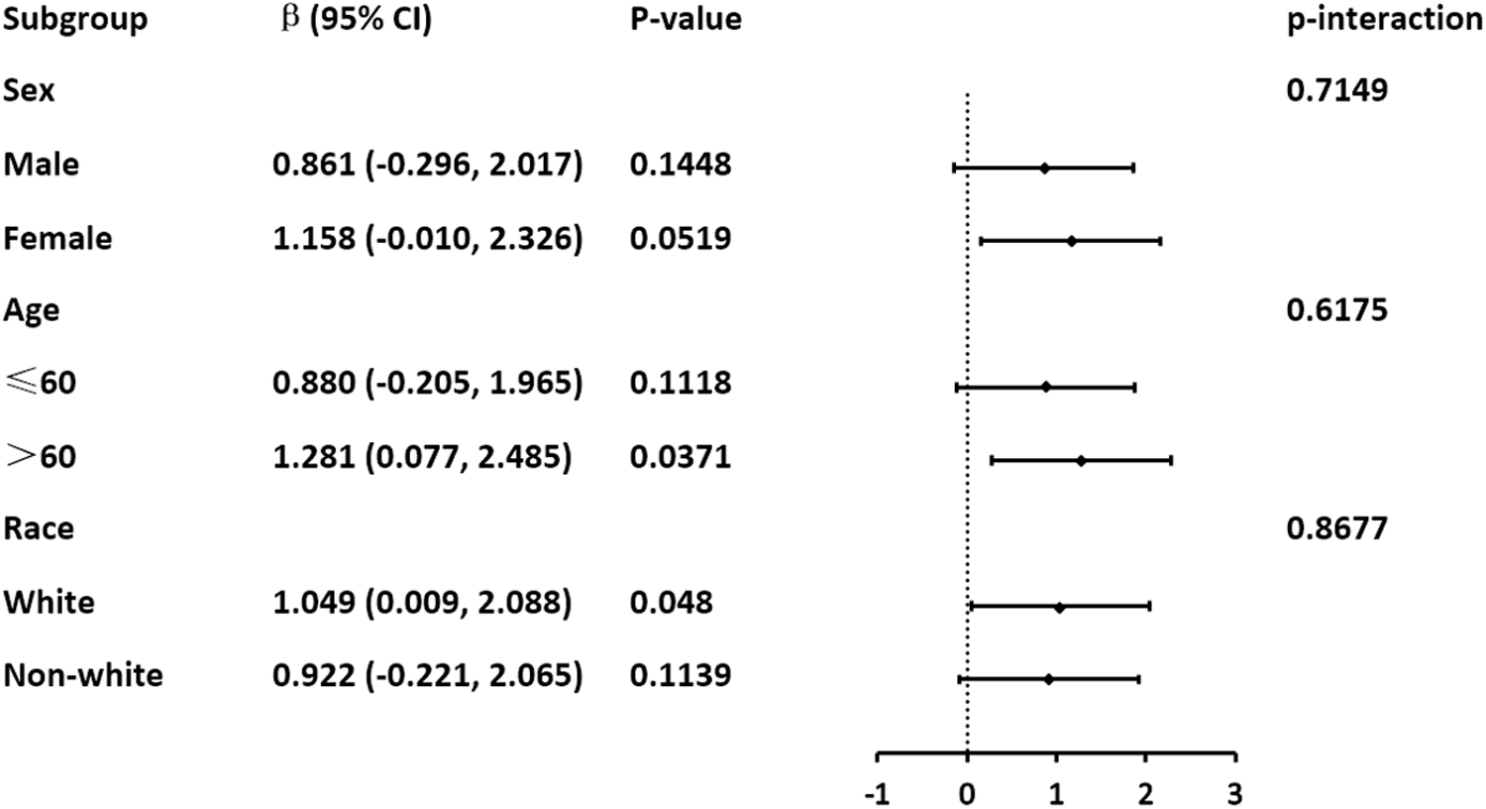
Forest plots of stratified analyses of Healthy Eating Index (AHEI)-2010 and S-Klotho.

To further verify whether this relationship still exists in different populations, we conducted subgroup analyses for age, gender, and race. Age was divided by a cutoff of 60 years, race was grouped by whether it was non-Hispanic White people, and we calculated the β values, confidence intervals, and P values for each subgroup. To better illustrate this relationship, we plotted Fig 3 for display. After adjustment using the Bonferroni correction method, that is, with the P-value adjusted to 0.05/3 = 0.0167, the P-values for all subgroup analyses were greater than 0.05 across different populations. We calculated the p for interaction for each subgroup, all of which were greater than 0.05, indicating no interaction between the AHEI and gender, age, and race, hence the results of each grouping are reliable.

## 4. Discussion

To the best of the author’s knowledge, this study is the first large-scale cross-sectional study to investigate the relationship between the Alternate Healthy Eating Index (AHEI) and S-Klotho. Through linear regression, we found a positive correlation between the AHEI score and S-Klotho levels in the middle-aged and elderly population in the United States, meaning that S-Klotho levels increase as the AHEI score rises. This positive correlation still exists after adequate adjustment. After quartile grouping. of the AHEI, the regression models also confirmed that S-Klotho levels increase with a higher AHEI quartile (p for trend < 0.05). Further curve fitting revealed a positive linear correlation between the two. In the stratified analysis, the P for interaction values were all  > 0.05, indicating that there is no significant difference in this relationship across different subgroups.

The AHEI score is composed of various components, and there are existing studies that explore the relationship between the intake of individual or a few components and S-Klotho levels. Vegetables, fruits, whole grains, nuts and legumes are all rich in fiber. Liu S [16] and colleagues found a positive correlation between dietary fiber intake and S-Klotho levels in the American population. Yang W and others discovered in a mouse experiment that the intake of N-3 polyunsaturated fatty acids can increase the expression of S-Klotho [17]. Regarding alcohol, sugar-sweetened beverages and fruit juices, Ostojic SM et al. [18], through NHANES, studied the relationship between various dietary elements and S-Klotho levels and found that a higher intake ratio of carbohydrates, total sugars, and a lower intake ratio of alcohol are positively related to S-Klotho levels. For trans fatty acids, Liang Y and colleagues found an L-shaped relationship with Klotho levels in the American population, with a negative correlation before the inflection point and a positive correlation after the inflection point [19]. Regarding sodium intake, Hu J-W and others found in the Chinese population that low-sodium diets can increase S-Klotho levels, while high-sodium diets reduce S-Klotho levels [20]. There are no articles studying the relationship between the intake of components such as long-chain omega-3 fatty acids and red and processed meats with S-Klotho levels. These all suggest a possible correlation between the AHEI and S-Klotho.

Regarding the relationship between diet patterns and S-Klotho levels, there are already relevant reports. Ma T-C et al [21]analyzed the relationship between the Healthy Eating Index (HEI-2015) and S-Klotho levels through the NHANES database and found that the higher the HEI-2015, the higher the S-klotho levels. Xiang L et al [22] investigated the correlation between Dietary Carbohydrate Intake and S-Klotho levels, discovering an Inverse J-Shaped relationship between the two. Ma T-C et al. [23]studied the relationship between the dietary inflammatory index and S-Klotho in the American middle-aged and elderly population, finding that the higher the inflammatory index, the lower the S-Klotho levels. Wu S-E and colleagues utilized the NHANS database and found the positive relationship between the Mediterranean Diet and klotho. Further component analysis revealed that the components affecting s-klotho levels included alcohol consumption, fruit, and dairy products[24].These studies indicated that a healthy diet can delay aging and maintain health.

However, there is currently a lack of literature on the relationship between AHEI and S-Klotho. Although both the HEI and AHEI emphasize increasing the intake of vegetables, fruits, and whole grains, and reducing the intake of sodium, added sugar, and saturated fat, the AHEI-2010 pays more attention to include foods and nutrients that can lower the risk of chronic diseases. Compared to HEI, AHEI includes alcohol consumption. In the study by Chiuve SE et al [10]., both are related to coronary heart disease, diabetes, stroke, and hypertension, but the association of the AHEI index is stronger. The relationship between AHEI and coronary heart disease, and diabetes does not change with the adjustment of the HEI index. However, the relationship between the HEI index and coronary heart disease, and diabetes disappears after the results are adjusted for AHEI. This demonstrates that the association between AHEI and chronic diseases is more robust. Therefore, we speculate that there should be a positive correlation between AHEI and S-Klotho levels, and our study has confirmed this hypothesis.

The potential mechanisms underlying the relationship between AHEI and S-Klotho may be associated with the lower pro-inflammatory effects of a healthy diet and lower levels of oxidative stress. Piccand E et al. [25] studied the relationship between the AHEI score and inflammatory biomarkers, finding a negative correlation between the AHEI score and C-reactive protein. Li SX et al. [26] conducted a cohort study and found a negative correlation between AHEI and inflammatory factors such as kynurenines, neopterin, IFN-γ, cytokines, and C-reactive protein. Crawford B et al. [27] in their cohort study discovered a negative correlation between the AHEI score and oxidative stress. Oxidative stress and inflammation can influence the expression of Klotho protein. Mitobe M and colleagues discovered that oxidative stress diminishes Klotho protein expression in a mouse kidney cell line [28]. This relationship has also been observed in human cell experiments, and it has been confirmed that it is mediated by the overexpression of microRNA-200c, which suppresses Klotho expression [29]. In terms of inflammatory cytokines, TNF-α and the TNF-like weak inducer of apoptosis suppress the expression of Klotho by activating NFκB-related signaling pathways [30]. Additionally, C-C motif chemokine 5 downregulates Klotho expression through the CCL5/p-STAT3/DNMT1 axis [31]. Overall, a high AHEI score may potentially elevate Klotho levels by reducing the levels of inflammatory cytokines. Secondly, it may be related to its role in reducing the risk of chronic diseases. The AHEI score has been found to be associated with the risk of chronic diseases such as coronary heart disease, diabetes, stroke, and hypertension[10]. Thirdly, it may be related to metabolic syndrome. Al Kudsee K et al. [32] found a negative correlation between AHEI and the incidence of metabolic syndrome, which in turn has a significantly negative correlation with S-Klotho [33]. Additionally, there may be other mechanisms at play, which require further research for clarification.

Due to the large number of participants included, we conducted subgroup analyses based on age, gender, and race. The study results indicate that the significant relationship between the AHEI index and S-Klotho is maintained in the age older than 60 years old and non-Hispanic White subgroups. The specific mechanisms are currently unclear, but it is hypothesized that this may be related to dietary and metabolic differences among different populations.

Although our study had endeavored to include factors that may influence the relationship between the AHEI index and S-Klotho, there are still elements such as comorbid conditions, prescription medications, and genetic predispositions that could potentially impact this association. S-Klotho levels are downregulated in a variety of diseases, including renal diseases, Alzheimer’s disease, chronic obstructive pulmonary disease, certain tumors, and vascular diseases [34–38]. These factors were not included as confounders in this paper due to the limited number of affected individuals in our study population and the incomplete understanding of the relationship between these diseases and S-Klotho levels. Similarly, some medications may upregulate Klotho levels, such as infliximab, empagliflozin, sulodexide, pioglitazone, and others [39]; however, there is a scarcity of related studies and the evidence is of low grade, which is why they were not considered as confounding factors. Regarding genetic predispositions, there is a lack of large-scale relevant studies. Additionally, our study included different populations as subgroup analyses, and the results showed differences among various ethnicities. This suggests that genetic factors may influence Klotho levels and further research is needed to elucidate this relationship.

Our research demonstrates a positive correlation between the AHEI score and S-Klotho levels. In the American population, if S-Klotho levels are found to be low, a healthy diet can be assessed through the AHEI, allowing for early intervention to delay the aging process. This has significant implications for the allocation of medical resources. Targeted adjustments for populations with low AHEI levels could potentially reduce the progression of aging and decrease the associated societal burden.

Our study has several limitations. First, our research is a cross-sectional study, so although the study concludes that there is a positive correlation between the AHEI score and S-Klotho levels, the causal relationship between the two cannot be determined and requires further research to clarify. Second, the study population is limited to the American population, and whether it can be further extended to other populations requires further research. Third, the study population is restricted to the elderly population over the age of 40, and further research is needed for adolescents. Fourthly, as the AHEI scores in this paper are based on self-reported dietary data, which are susceptible to recall bias and inaccuracies, these factors may potentially influence the conclusions drawn from this study. We hope that future research with more detailed methodologies will address these biases. Despite these, our study is a large-sample clinical study based on complex sampling and is also the first study to investigate the relationship between AHEI and S-Klotho, providing a strong research foundation for the relationship between diet and S-Klotho. It also suggests that for people with low S-Klotho levels (especially elderly non-Hispanic White people), their dietary quality should be assessed through AHEI to delay the onset of aging.

In conclusion, AHEI is positively associated with S-Klotho levels in middle-to-older American adults. Further research is needed to elucidate the causal relationship and specific mechanisms.
